# Contrast enhanced ultrasound of liver lesions in patients treated for childhood malignancies

**DOI:** 10.1186/s40644-024-00750-3

**Published:** 2024-08-29

**Authors:** Ayatullah G. Mostafa, Zachary Abramson, Mina Ghbrial, Som Biswas, Sherwin Chan, Himani Darji, Jessica Gartrell, Seth E Karol, Yimei Li, Daniel A. Mulrooney, Tushar Patni, Tarek M Zaghloul, M. Beth McCarville

**Affiliations:** 1 Department of Diagnostic Imaging, St. Jude Children’s Research Hospital MS 220, 262 Danny Thomas Place, Memphis, TN 38105 USA; 2 Department of Diagnostic Imaging, Cairo University, Cairo, Egypt; 3 Pediatric Radiology Department, Le Bonheur Children’s Hospital, Memphis, TN USA; 4 Department of Radiology, Children’s Mercy Hospital, 2401 Gillham Rd, Kansas City, MO 64108 USA; 5 Department of Biostatistics , St. Jude Children’s Research Hospital, 262 Danny Thomas Place, Memphis, TN 38105 USA; 6 Department of Oncology, MS 260, St. Jude Children’s Research Hospital, 262 Danny Thomas Pl, Memphis, TN 38105 USA; 7 Department of Surgery, MS 133, St. Jude Children’s Research Hospital, 262 Danny Thomas Place, Memphis, TN 38105 USA; 8 Department of Surgery, National Cancer Institute, Cairo University, Cairo, Egypt

**Keywords:** Contrast enhanced ultrasound, Focal liver lesions, Pediatric malignancy

## Abstract

**Background:**

Patients treated for cancer have a higher incidence of focal liver lesions than the general population and there is often concern for a malignant etiology. This can result in patient, caregiver and physician anxiety and is managed by a “wait and watch” approach, or immediate additional imaging, or biopsy, depending on the degree of clinical concern. Because it is a low-cost, easily accessible, radiation and sedation free modality, we investigated the value of contrast enhanced ultrasound (CEUS) to accurately distinguish benign from malignant liver lesions in patients treated for childhood malignancies.

**Methods:**

We performed an IRB approved retrospective study of 68 subjects who were newly diagnosed, on treatment or off treatment for a pediatric malignancy and had liver lesions discovered on CT, MRI or non-contrast ultrasound and subsequently underwent CEUS between September 2013 and September 2021. Two experienced pediatric radiologists and a radiology trainee, blinded to the etiology of the liver lesions, independently reviewed the CEUS examinations and categorized lesions as benign, indeterminate, or malignant. The reference standard was biopsy for 19 lesions and clinical follow-up for 49. The sensitivity, specificity, positive and negative predictive value, and diagnostic accuracy of CEUS were calculated using only the benign and malignant CEUS classifications. Inter-reviewer agreement was assessed by Cohen’s kappa statistic.

**Results:**

There were 26 males and 42 females, mean age, 14.9 years (range, 1–52 years). Fifty subjects were off therapy, twelve receiving treatment, and six with newly diagnosed cancer. By the reference standard, 59 (87%) lesions were benign and 9 (13%) were malignant. Sensitivities of CEUS for the three reviewers ranged from 83 to 100% (95% CI, 35.9-100%), specificities from 93.1 to 96.0% (95% CI, 83.5-99.6%), PPV 60.0-71.4% (95% CI, 29.0-96.3%), NPV 98.0-100% (95% CI, 89.2-100%) and accuracy from 93.8 to 94.6% (95% CI, 85.1-99.7%). The kappa statistic for agreement between the two experienced radiologists was moderate at 0.58.

**Conclusions:**

CEUS is highly accurate in distinguishing benign from malignant etiologies of liver lesions in patients treated for pediatric malignancies.

## Background

The incidence of focal liver lesions (FLLs) in pediatric and adult cancer patients after cessation of treatment is higher than in the general population [1–5]. It is hypothesized that multi-agent chemotherapy is a risk factor for focal hepatic circulatory disturbances. It is believed that, in this setting, arterial and portal venous thrombosis leads to the development of benign hepatic regenerating lesions secondary to vascular recanalization, reperfusion and hepatocyte proliferation [6–8]. Additionally, chemotherapy and radiation therapy may cause injury to the vascular endothelium [9] that could impair local perfusion. Such lesions are often referred to as focal nodular hyperplasia-like lesions (FNH-like) because they are similar to the classic, benign FNH that occur in normal liver, but occur in the presence of underlying liver disease [10]. In a review of 273 children treated for solid malignancies, Smith et al. found that at 2 or more years from diagnosis, 17% (46/273) developed FLLs on surveillance imaging using CT or MRI. When liver lesions arise in patients treated for cancer, there is often a concern for metastatic disease and additional testing is required. Several approaches may be undertaken. One is to “wait and watch” with repeat imaging at 6 to 8 weeks to determine the growth rate of the lesion. However, this results in patient, caregiver and treating physician anxiety and may delay treatment of a malignant process. Another approach is to promptly reimage using a different modality, such as hepatocyte specific MRI or CT, to further characterize the lesion. This adds to the cost of medical care and may necessitate the use of sedation or anesthesia or expose the patient to the harmful effects of radiation. Radiation and sedation exposure are of particular concern in the pediatric oncology population because patients typically undergo numerous exposures before, during and after treatment and children have a longer life expectancy in which to develop a radiation induced cancer. Furthermore, repetitive sedation during childhood has been linked to greater neurocognitive impairment after completion of therapy [11] and should be avoided whenever possible. Finally, depending on the clinical scenario and index of suspicion, an invasive procedure, such as biopsy, may be performed. Additionally, pediatric patients may present de novo with primary liver lesions of uncertain etiology including congenital hemangioma, hepatoblastoma, and classic FNH. The ideal management of these patients would be a non-invasive, low-cost, radiation and sedation free modality with a high sensitivity and specificity for distinguishing the benign or malignant nature of the lesion. Contrast enhanced ultrasound (CEUS) has been shown to be a reliable method of distinguishing benign from malignant liver lesions in the general adult and pediatric population. However, there are no reports of the value of CEUS specifically in a population of patients who have a newly diagnosed pediatric malignancy or are undergoing or have completed treatment of a childhood cancer. The purpose of our study was to assess the utility of CEUS and determine the sensitivity, specificity, positive and negative predictive value, and diagnostic accuracy for distinguishing benign from malignant liver lesions in this patient population. We also assessed the inter-reviewer agreement and potential impact of length of experience with CEUS to correctly categorize liver lesions by comparing the findings of a radiology trainee to two more experienced radiologists.

## Methods

Waiver of consent for this single-center retrospective study was approved by our IRB. Our large pediatric cancer hospital conducts an extensive after completion of therapy program that follows patients into adulthood to determine the long-term effects of childhood cancer therapy. Over the past ten years we have routinely recommended CEUS when liver lesions are identified on other imaging modalities in newly diagnosed patients, those receiving treatment and after completion of therapy. Using our radiology informatics system, we identified 71 subjects with liver lesions who underwent 84 CEUS examinations between September 2013 and September 2021. Thirteen subjects had more than one CEUS for follow-up of the same liver lesion; we included only the first examination for purposes of this study. Three examinations were excluded due to technical failure. The final cohort included 68 CEUS examinations in 68 patients. We recorded the modality (US, CT, or MRI) and type of exam (i.e., spine MRI, single phase contrast enhanced abdomen CT etc.) by which the lesion was first discovered and whether there was a solitary lesion or multiple lesions. For purposes of CEUS an index lesion was chosen based on ease of visibility on US (see below). From the medical record we recorded demographics, primary cancer diagnosis and whether the subject was newly diagnosed, on-therapy or off-therapy at the time of CEUS. For those who were off therapy, the time from completion of therapy to discovery of the liver lesion was recorded.

From September 2013 through November of 2018 all CEUS examinations were performed on a GE LOGIQE9 ultrasound scanner and from December 2018 through September 2021 exams were performed on either a LOGIQE9 or LOGIQE10 scanner (General Electric Healthcare, Milwaukee, WI, USA). Depending on the patient’s body habitus, either a curvilinear 4–6 MHz or a linear 9 MHz transducer was used. Pre-contrast US was performed to identify the patient position and sonographic window that allowed optimal visualization of the index lesion. In general, the index lesion was chosen based on ease of visibility including adequate acoustic window, size (larger lesions preferred) and distance from diaphragm (farther from diaphragm preferred to minimize breathing motion). The largest diameter of the index lesion was documented on grayscale images. All CEUS exams were performed with a mechanical index ≤ 0.3. Sixty-one were performed with a perflutren contrast agent (GE Healthcare, Princeton, NJ) using a dose of 0.3 mL for patients < 20 kg and 0.5 mL for those ≥ 20 kg. Seven were performed with a sulfur hexafluoride lipid contrast agent (Bracco Group, Milan, Italy) using 0.03 mL/kg. Contrast was given through a central or peripheral venous line followed by 10 ml normal saline flush. A radiologist was present for all examinations and repeat injections were given if deemed necessary by the radiologist. The number of contrast injections and immediate reaction to the contrast material, if present, were recorded. According to previously published good clinical practice guidelines [12], we recorded continuous, dynamic imaging of the index lesion for one minute and then intermittent imaging up to 5 min after injection, to allow characterization of the lesion in the arterial, portal-venous and delayed phases of enhancement. Immediately following the 1-minute recording of the index lesion, we also obtained cine clips during sweeps through the remainder of the liver in the transverse and longitudinal planes to assess any additional lesions.

There were three study radiologists, one pediatric radiologist with 18 years of CEUS experience at our pediatric cancer center (MBM), one pediatric radiologist with about 4 years of CEUS experience (ZA) at our pediatric cancer center and a pediatric radiology trainee with no prior CEUS experience (SB) at the start of the study. The pediatric radiology fellow was trained by the more experienced radiologist (MBM) in interpretation of CEUS of liver lesions for purposes of this study. Training consisted of a review of the literature followed by an “at the console” concurrent review and discussion of 10 recent cases that were not included in the study cohort. This was followed by an independent review of 10 additional, non-study cases by the trainee who gave interpretations of liver lesions as benign, malignant, or indeterminate. Because there was 100% concordance in independent interpretations between the trainer and trainee, the trainee was deemed qualified to interpret CEUS for purposes of this study.

To assess the impact of clinical information on the interpretation of CEUS the study radiologists reviewed the imaging twice. During both reviews the radiologists were blinded to the final diagnosis of each liver lesion as determined by the reference standard described below. The first review was performed without knowledge of the patient’s primary tumor diagnosis or clinical history and will be referred to as the “blinded” review. The second review was performed with full knowledge of the patient’s primary tumor diagnosis and clinical history at the time of the CEUS exam and will be referred as the “unblinded” review. To mitigate potential recall bias, we allowed at least two months between the performance of the clinical CEUS and reviews. Based on prior reports describing the CEUS features of benign and malignant liver lesions, the study radiologists defined lesions as benign if they did not demonstrate washout in any phase, malignant if they demonstrated washout in the portal venous or delayed phase and indeterminate if there was only subtle washout that was difficult to confirm or the enhancement pattern was difficult to ascertain [13–18]. The pattern of lesional enhancement (hypoenhancing, isoenhancing or hyperenhancing compared to normal liver) in the three phases was recorded by the more experienced study radiologist (MBM). During the “unblinded” reviews, the study radiologist’s indeterminate interpretations included a subjective component based on clinical experience and took into consideration clinical circumstances such as the index of suspicion for malignancy. Results from biopsy, when available, or clinical follow-up, including additional imaging and management decisions, were used as the reference standard for the final benign vs malignant designation of the liver lesions.

### Statistical analysis

Descriptive statistics are provided to summarize the study population. For categorical variables, numbers and percentages are provided. For continuous variables, mean, standard deviation, median and range were calculated. After excluding indeterminate categorizations, the sensitivity, specificity, positive predictive value (PPV), negative predictive value (NPV), and diagnostic accuracy of the CEUS interpretations by the three reviewers for the the “blinded” and “unblinded” reviews were estimated along with their 95% confidence intervals. Cohen’s kappa statistic was used to assess inter-reviewer variability between the two experienced reviewers (MBM, ZA) and between the more experienced reviewer (MBM) and the pediatric radiology trainee (SB). Analyses were performed with SAS 9.4 and R version (4.1.2).

## Results

There were 26 males and 42 females, mean age, 14.9 years (range, 1–52 years); 49 white (49/68, 72.1%), 14 black (14/68, 20.6%), 4 Asian (4/68, 5.9%), and 1 mixed race (1/68, 1.5%). Thirty-two of the liver lesions were first discovered on CT (3 on CT bone density studies, 3 on non-contrast enhanced chest CT, 5 on contrast-enhanced chest CT, 1 on non-contrast enhanced abdomen, 20 on single-phase contrast enhanced abdominal CT), 24 on MRI (5 detected on liver iron MRI, 2 on spine MRI, 2 on hepatocyte specific contrast enhanced abdominal MRI, 15 on conventional contrast enhanced abdominal MRI), and 12 on non-contrast enhanced US. Thirty-two subjects (32/68, 47.1%) had solitary lesions and 36 (36/68, 53.0%) had multiple lesions, ranging in number from 2 to > 10. Primary cancer diagnoses are shown in Table 1.

**Table 1:** Primary cancer diagnosis of 68 subjects undergoing CEUS of focal liver lesions

Primary Cancer Diagnosis (*n*)	Number of patients (%)
Leukemia AML (5) ALL (5) Mixed phenotype leukemia (1)	11 (16.2%)
Neuroblastoma	10 (14.7%)
Medulloblastoma	7 (10.2%)
Lymphoma Hodgkin lymphoma (3) Large B cell lymphoma (1) NHL T-lymphoblastic lymphoma (1) Diffuse high-grade, B cell lymphoma (1)	6 (8.8%)
Ewing sarcoma	5 (7.4%)
Wilms tumor	5 (7.4%)
Chondrosarcoma	3 (4.4%)
Infantile fibrosarcoma	3 (4.4%)
Hepatoblastoma	3 (4.4%)
Hepatocellular carcinoma	2 (2.9%)
Desmoplastic small round cell tumor	2 (2.9%)
Osteosarcoma	2 (2.9%)
Gastric carcinoid, adrenocortical carcinoma, rhabdomyosarcoma, yolk sac tumor, glioblastoma, hemangiopericytoma, malignant peripheral nerve sheath tumor, ocular melanoma, retinoblastoma.	1 each (1.5%; 13.5% total)

Based on grayscale US measurements obtained at the time of the CEUS, there was substantial overlap in the size of the index benign, indeterminate, and malignant lesions although malignant lesions tended to be larger; benign lesions ranged from 0.60 to 7.1 cm (mean 2.19 cm, SD 1.37), indeterminate from 0.50 to 4.90 (mean 2.03, SD 1.96) and malignant from 0.70 to 16.50 cm (mean 4.35, SD 5.70). During CEUS examination, eight patients required second injections to improve visualization or characterization of the index lesion. One patient reported altered taste after administration of the perflutren contrast agent. There were no other adverse reactions to the ultrasound contrast material.

Fifty subjects (50/68, 73.5%) were diagnosed with liver lesions while off therapy, 12 during treatment (12/68, 17.7%), and six (6/68, 8.8%) at the time of primary cancer diagnosis. Nineteen biopsies were performed in 19 subjects and used as the reference standard. The median time between biopsy and CEUS was 0.43 months with a range of 0–7.5 months. Clinical follow-up served as the reference standard in the remaining 49. The median length of clinical follow-up was 2 years and 6 months with a range of 0 months to 7 years and 10 months. Final diagnoses of liver lesions were 59 benign (14 biopsied) and 9 malignant lesions (5 biopsied). Among the 50 subjects who were off therapy, 48 (48/50, 96%) had benign lesions and 2 (2/50, 4%) had malignant lesions. The two with malignant lesions were a Wilms tumor recurrence 7 months off therapy, and neuroblastoma recurrence in a 4-month-old, 6 weeks after surgical treatment alone. Mean time from completion of therapy to discovery of liver lesions was 5.72 years (range 0 to 33 years). Among 12 lesions discovered in subjects who were on therapy, 2 were malignant and 10 benign. The 2 malignant lesions were both due to metastatic disease, one in a patient with adrenocortical carcinoma and one with desmoplastic small round cell tumor. Among the 7 subjects with liver lesions at the time of cancer diagnosis, 2 had hepatoblastoma, 1 had hepatocellular carcinoma, 2 had Wilms tumor with hepatic metastases, and 2, one with Hodgkin lymphoma and one with fibrosarcoma, had benign lesions with CEUS features suggestive of FNH (central early vessels in spoke wheel pattern, centrifugal enhancement, no delayed washout).

The sensitivity, specificity, PPV, NPV, and diagnostic accuracy, calculated by excluding indeterminate categorizations, of the “blinded” and “unblinded” reviews for the three study radiologists are shown in Table 2. The unblinded reviews showed excellent specificity (≥ 93.1%), NPV (≥ 98.0%) and accuracy (≥ 93.8%) for all three reviewers.

**Table 2:** Sensitivity, specificity, positive predictive value, negative predictive value, and diagnostic accuracy of CEUS interpretations for distinguishing the benign vs. malignant nature of FLLs compared to the reference standard for 2 experienced study radiologists and a radiology trainee when blinded to clinical history and unblinded to clinical history (except the lesional diagnosis)

	Blinded to Clinical Information			Unblinded to Clinical Information (exept lesional diagnosis)		
	Experienced Radiologist Reviewer 1 (95% CI)	Experienced Radiologist Reviewer 2 (95% CI)	Radiology Trainee (95% CI)	Experienced Radiologist Reviewer 1 (95% CI)	Experienced Radiologist Reviewer 2 (95% CI)	Radiology Trainee (95% CI)
**Sensitivity**	77.8% (50.2–100)	85.7% (42.1–99.6)	0% (0–84.2)	100.0% (100–100)	100% (54.1–100)	83.3% (35.9–99.6)
**Specificity**	86.8% (77.7–95.9)	92.2% (81.1–97.8)	91.5% (79.6–97.6)	93.1% (86.6–99.6)	94.0% (83.5–98.8)	96.0% (86.3–99.5)
**Positive Predictive Value**	50.0% (23.8–76.2)	60.0% (26.2–87.8	0% (0–60.2)	60.0% (29.4–90.4)	66.7% (30-92.5)	71.4% (29.0–96.3)
**Negative Predictive Value**	95.8% (90.2–100)	97.9% (88.9–100)	95.6% (84.9–99.5)	100.0% (100–100)	100% (92.5–100)	98.0% (89.2–100)
**Accuracy**	85.5% (76.7–94.3)	91.4 (81-97.1)	87.8% (75.2–95.4)	93.8% (87.8–99.7)	94.6% (85.1–98.9)	94.6% (85.1–98.9)

**Fig. 1:**
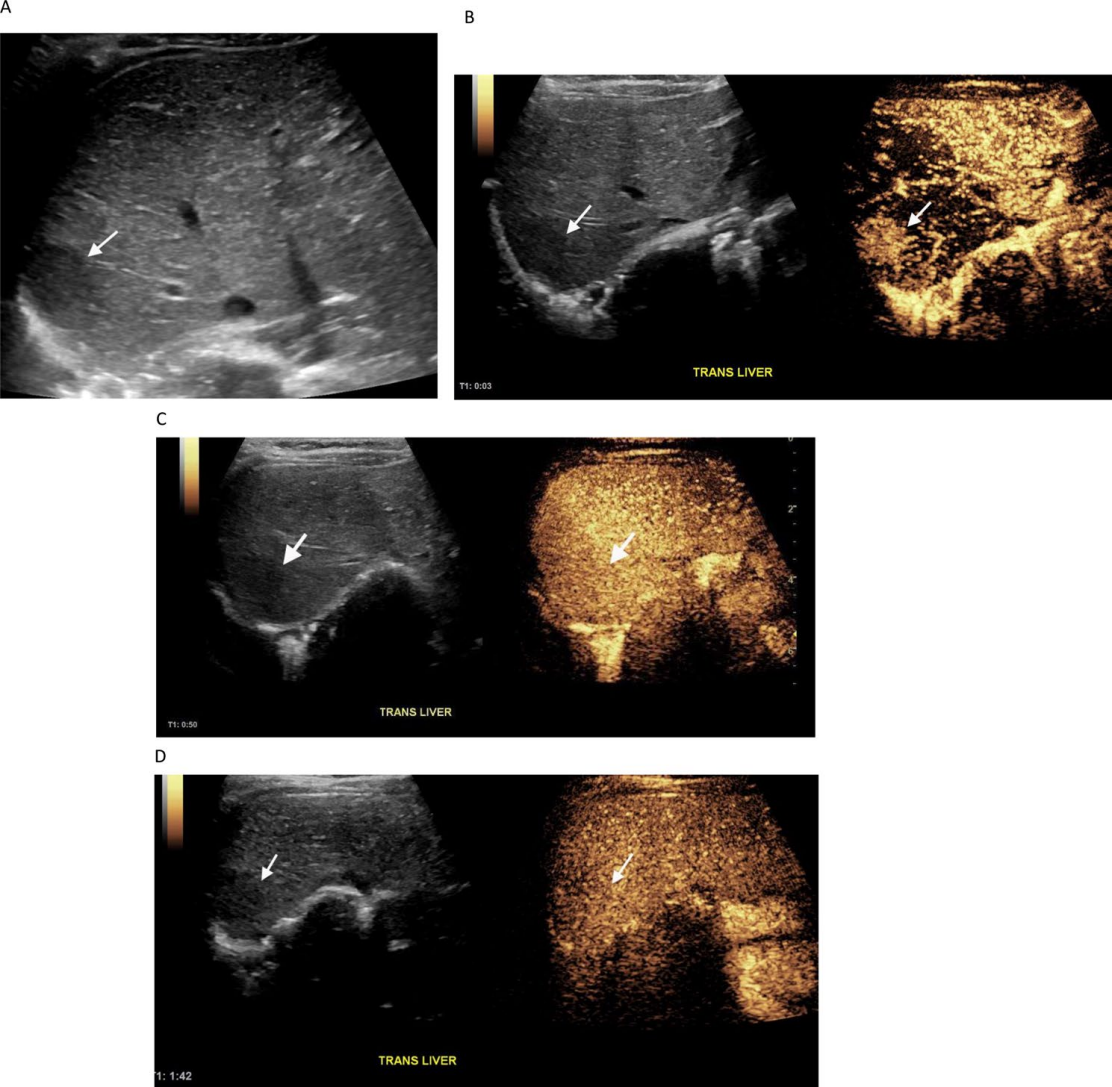
5-month-old female with Trisomy 18 had an (**A**) Incidentally discovered FLL (arrow) on surveillance ultrasound (**B**) Arterial-phase CEUS image obtained 3 s after contrast administration shows hyperenhancement of the lesion (arrows). (**C**) Portal-venous-phase CEUS image obtained at 50 s after injection and (**D**) delayed-phase CEUS image obtained at 1 min and 42 s both show isoenhancement of the lesion (arrows). The lesion grew on two-month follow-up and was pathologically proved to be hepatoblastoma

**Fig. 2:**
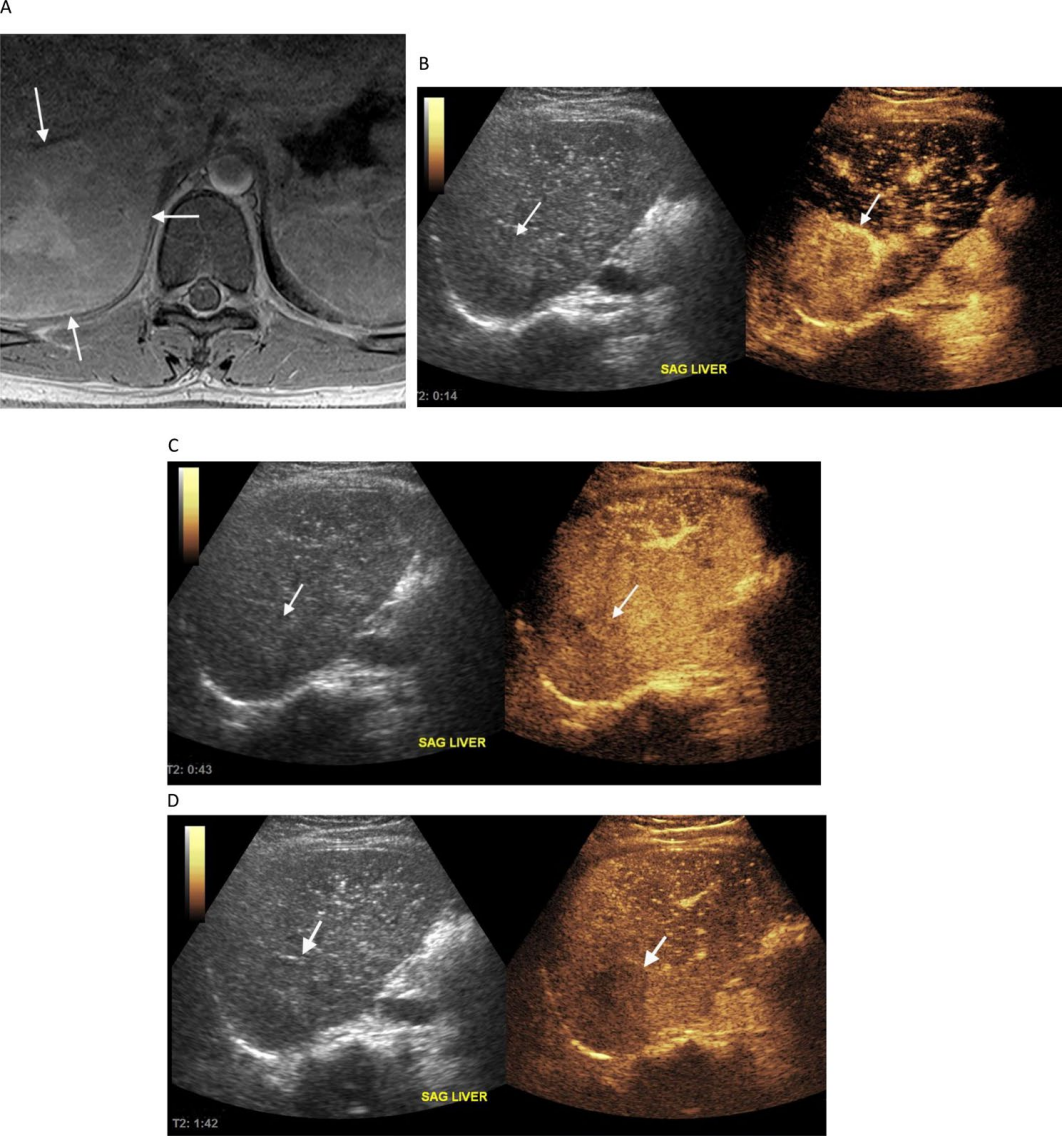
18-year-old male, medulloblastoma survivor, 12.5 years after completion of therapy, developed a focal liver lesion (FLL) that was incidentally discovered as (**A**) heterogeneity within the liver (arrows) on T1W contrast enhanced axial spine MRI. (**B**) Arterial-phase contrast enhanced ultrasound (CEUS) image obtained 14 s after contrast administration shows hyperenhancement of the lesion (arrows). (**C**) Portal-venous-phase CEUS image obtained at 43 s and (**D**) delayed-phase CEUS image obtained at 1 min 42 s after injection show hypoenhancement of the lesion. This was pathologically proved to be focal nodular hyperplasia

**Fig. 3:**
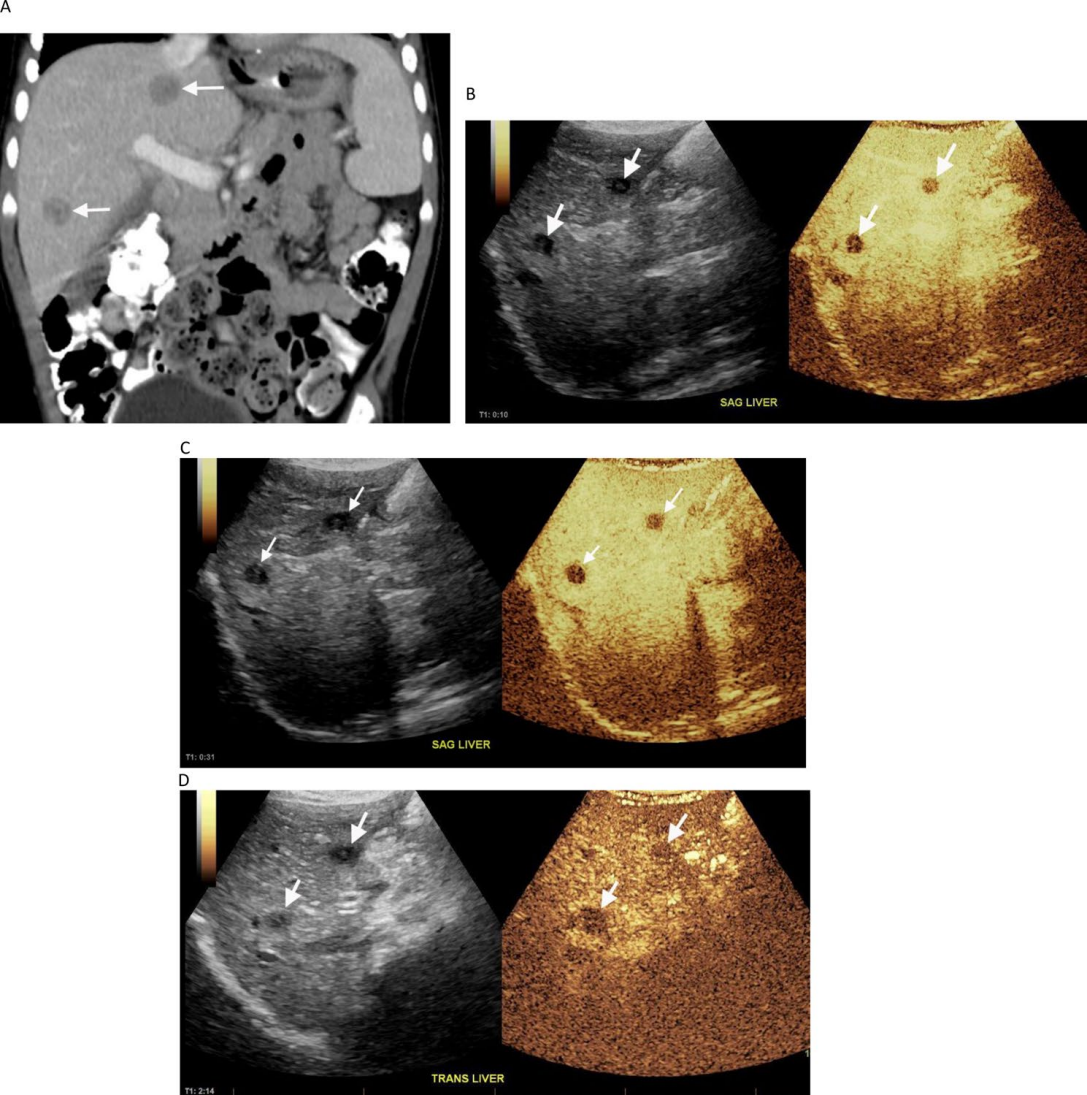
2-year-old male with a presacral yolk sac tumor developed multiple FLL’s while on therapy. (**A**) Coronal contrast enhanced CT image shows two well defined hypodense lesions (arrows). (**B**) Arterial-phase CEUS image obtained at 10 s after contrast administration shows two hypoenhancing lesions (arrows). (**C**) Portal-venous-phase CEUS image obtained at 31 s after injection and (**D**) delayed-phase CEUS image obtained at 2 min and 14 s after injection again show hypoenhancement of the lesions (arrows). These were pathologically proven to be abscesses with necrotizing granuloma

When considering the “unblinded” reviews by the more experienced radiologist (MBM) there was agreement with the reference standard in 60/68 (88.2%) cases, with 4 indeterminates and 4 false positives. All 4 indeterminate and 3 of 4 false positives were biopsied. Among the indeterminates, one was metastatic adrenocortical carcinoma in a 3 year old patient with Li-Fraumeni syndrome, one was a 19 month old patient with Beckwith Wiedemann syndrome and hepatoblastoma,, one was hepatoblastoma in a 5-month-old patient with trisomy 18 (Fig. 1), and one was an FNH-like lesion in an 18 year old patient with a central nervous system malignancy who was 12.5 years off therapy and considered to be at low risk for metastatic recurrence (Fig. 2). Among the three biopsied false positives, one was a focus of cytomegalovirus infection, one was hepatocytes with hemosiderin deposition and one was lymphocytic and eosinophilic aggregates. A notable pattern of enhancement was present in a biopsied microabscess/necrotizing granuloma that was interpreted as benign by the more experienced radiologist (MBM), indeterminate by the less experienced radiologist (ZA) and malignant by the radiology trainee (SB). This lesion, shown in Fig. 3, was hypoenhancing in the arterial, portal venous and delayed phases. Patterns of contrast enhancement recorded by the more experienced study radiologist, the “unblinded” review interpretations for all three study radiologists with corresponding histology of the 19 biopsied liver lesions are shown in Table 3.

**Table 3:** CEUS enhancement pattern recorded by experienced study radiologist reviewer 1 and experienced radiologist reviewer 1, reviewer 2 and radiology trainee interpretations, and final histological diagnosis of 19 biopsied FLLs

CEUS enhancement pattern			Experienced Radiologist 1	Experienced Radiologist 2	Radiology Trainee	Histologic diagnosis
Arterial	Portal-venous	Delayed				
↑	↔	↓	Indeterminate	Malignant	Indeterminate	FNH
↑	↔	↔	Benign	Benign	Benign	Adenoma
↑	↔	↓	Benign	Indeterminate	Indeterminate	FNH
↑	↑	↑	Benign	Benign	Benign	FNH
↑	↔	↔	Benign	Indeterminate	Benign	Granulomatous inflammation
↑	↑	↑	Benign	Benign	Benign	Hepatocytes with Iron deposition
↔	↔	↔	Benign	Benign	Benign	Hepatitis with fibrosis and fungal infection
↑	↓	↓	Malignant	Malignant	Malignant	Metastatic Wilm’s tumor
↑	↓	↓	Malignant	Malignant	Malignant	Hepatocellular carcinoma
↔	↓	↓	Malignant	Malignant	Malignant	Hepatoblastoma
↓	↓	↓	Benign	Indeterminate	Malignant	Microabscess/ Necrotizing granuloma
↔	↓	↓	Malignant	Malignant	Indeterminate	CMV infection
↔ to ↑	↓	↓	Indeterminate	Malignant	Indeterminate	Metastatic adrenocortical carcinoma in patient with Li Fraumeni syndrome
↑	↓	↓	Malignant	Indeterminate	Indeterminate	Hepatocytes with iron deposition
↔ to ↓	↓	↓	Malignant	Indeterminate	Indeterminate	Lymphocytic and eosinophilic aggregates
↓	↓	↓	Indeterminate	Indeterminate	Malignant	Hepatoblastoma in patient with Beckwith Weidemann syndrome
↔ to ↑	↔	↔	Benign	Benign	Benign	FNH
↑	↔	↔	Indeterminate	Indeterminate	Benign	Hepatoblastoma in patient with trisomy 18
↑	↔	↓	Benign	Benign	Benign	Fatty change/possible FNH

↑ = hyperenhancement, ↔ = isoenhancement, ↓ = hypoenhancement

**Fig. 4:**
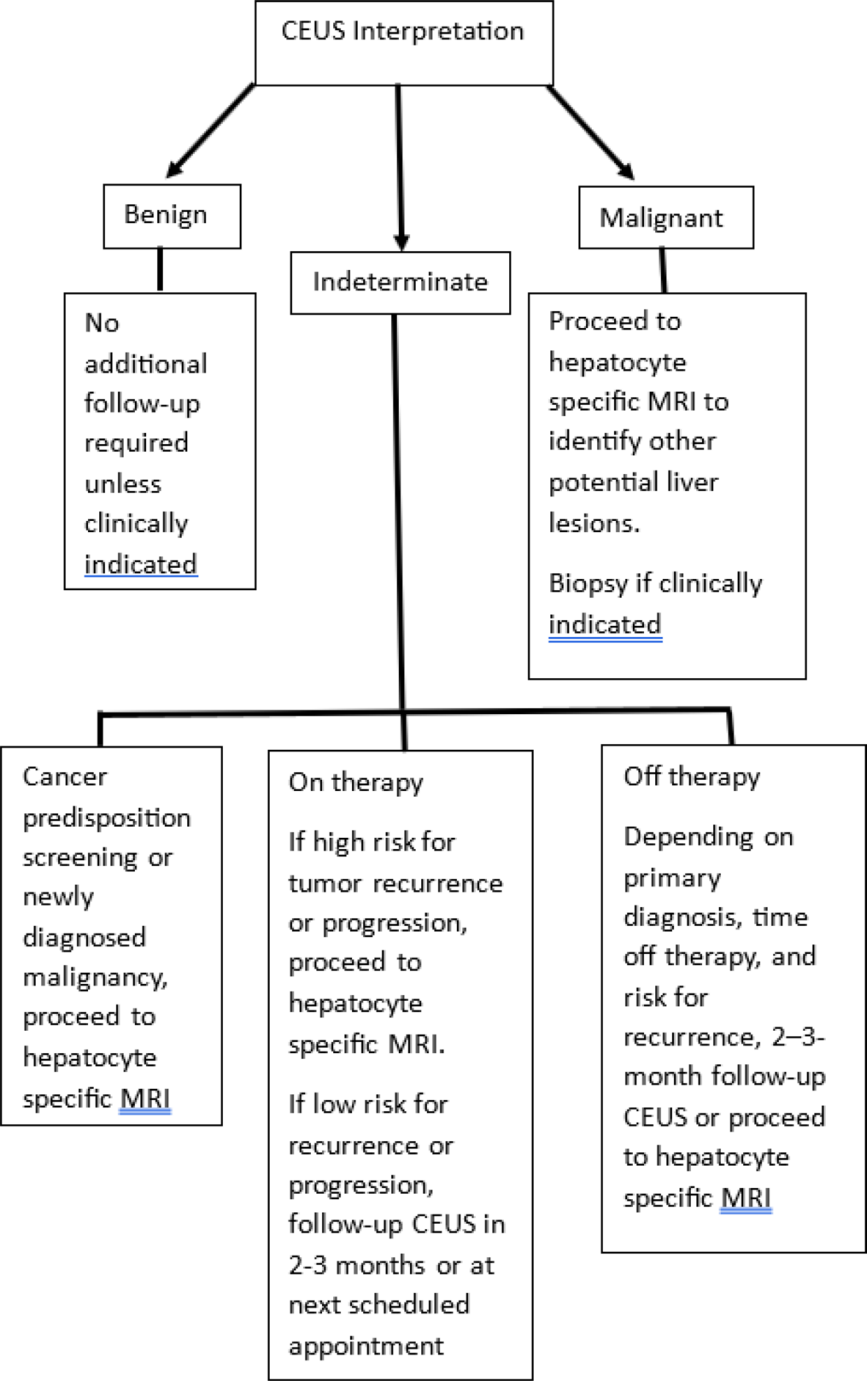
Proposed management algorithm for contrast enhanced ultrasound assessment of focal liver lesions in patients treated for pediatric malignancies

Table 4 shows the final reference standard, benign vs. malignant, diagnoses for indeterminate lesions for the unblinded reviews for all three reviewers. The more experienced radiologist had fewer indeterminates (*n* = 4) than the less experienced radiologist and radiology trainee (*n* = 12 for both). The kappa statistic for agreement between the more experienced and less experienced radiologist was 0.58, indicating moderate agreement, and between the more experienced radiologist and radiology trainee was 0.53, also indicating moderate agreement. A proposed clinical management algorithm based on CEUS findings and clinical features is provided in Fig. 4.

**Table 4:** Final reference standard benign vs. malignant diagnoses for indeterminate categorizations on “unblinded” reviews by 2 experienced radiologist reviewers and and the study radiology trainee

Reviewer	Indeterminates (*n*)	Reference standard (*n*)	
		Benign	Malignant
**Experienced Radiologist Reviewer 1**	4	1	3
**Experienced Radiologist Reviewer 2**	12	9	3
**Radiology Trainee**	12	9	3

## Discussion

We have shown that CEUS is a highly reliable method of distinguishing benign from malignant liver lesions in most patients treated for pediatric malignancies. Our findings add to the growing body of evidence that CEUS can replace other imaging modalities for this indication [19, 20]. A critical aspect in achieving a high degree of diagnostic accuracy with CEUS in the oncology setting is the need to consider the clinical scenario in which the examination is performed. This is evidenced in our study by the improved sensitivity, specificity, PPV, NPV and accuracy of the “un-blinded” reviews compared to the “blinded” reviews. We also found that the less experienced radiologists had more indeterminate classifications than the more experienced radiologist, which may have partially accounted for the moderate agreement between reviewers. This is notable since the study radiologists had the same clinical information available during the “unblinded” review. We attribute this to several factors. First, we acknowledge that there is a learning curve in the performance and interpretation of CEUS findings. Although not addressed by this study, our findings suggest that fewer indeterminate interpretations are made as radiologists gain more experience and confidence in the modality. In our study, this was coupled with inherent subject matter expertise of the more experienced study radiologist resulting from years of practice in a predominantly oncology setting compared to the less experienced radiologist and radiology trainee. This highlights a challenge to the generalizability of CEUS in the evaluation of incidental liver lesions especially in patients with a history of cancer. Knowledge of the biologic behavior of the primary malignancy is essential since some pediatric cancers can metastasize long after diagnosis while others rarely metastasize to the liver. We found that another important feature to consider is the likelihood of liver cancer (primary or metastatic) in children with cancer predisposition syndromes. The likelihood of infection should also be considered and may be reflected by laboratory values or presence of fever [21].

Perhaps most importantly, in the oncology setting, CEUS can help avoid the anxiety often associated with a “wait-and-watch” approach. At our institution when a liver lesion is discovered on routine imaging, CEUS is performed either the same or the next day. Because CEUS has high sensitivity, specificity, NPV and accuracy we can usually confidently report to the treating physician that the patient has a lesion that is highly likely to be benign or requires additional evaluation. This has led to a paradigm shift at our institution. Rather than bringing the patient back for early follow-up (wait and watch) or obtaining additional imaging with CT or MRI, we proceed directly to CEUS for a more cost-effective, sedation and radiation free evaluation that allows prompt and highly accurate results, improving overall clinical management.

The primary objective of our study was to determine if CEUS could accurately distinguish benign from malignant liver lesions, and we did not endeavor to further characterize the benign lesions. In the study by Smith et al. of 46 pediatric solid tumor patients who developed FLLs, 14 (36%) were classified as FNH (possibly FNH-like) either based on clinical and imaging findings (*n* = 10) or pathologic inspection (*n* = 4). Other benign etiologies included cysts, perfusion abnormalities and focal fat. In that study, the time to development of FNH (FNH-like) lesions was longer than for other benign etiologies and malignant lesions. Others have reported FNH (FNH-like) lesions among children at approximately 5.6 years after completion of antineoplastic and cytotoxic chemotherapy for a malignant tumor [22, 23]. In our study, 51 subjects (51/68, 75%) were treated for solid malignancies while 17 (17/68, 25%) had hematologic disease. Among the solid malignancies neuroblastoma (*n* = 10) and CNS tumors (*n* = 9) were the most common diagnoses. However, among all diagnoses, leukemia was the most common (*n* = 11). Although we cannot be certain of the etiology of many of the liver lesions in our study, we found that most were benign and the mean time to discovery was 5.72 years after completion of therapy. However, this finding should be interpreted with caution since without systematic intermittent imaging screening across the cohort we cannot be certain of the time point at which the liver lesions developed. It remains unclear whether there is a common inciting factor among various pediatric cancer diagnoses and treatments or if there are underlying predisposing factors to developing benign liver lesions in this cohort. Importantly, however, it should not be assumed that patients who develop liver lesions during long term follow-up after completion of therapy have only benign disease. Recurrent cancer in long term survivors of pediatric malignancies remains the number one cause of death [24], and ongoing worry about recurrent disease occurs in more than 25% of parents of childhood cancer survivors [25]. Therefore, the minimal time and cost of performing CEUS in this patient population is far outweighed by the benefit.

Perhaps the most informative findings in our study are the etiologies of biopsied indeterminate and false positive results on the “unblinded” reviews. Among the 4 indeterminates classified by the more experienced radiologist, 3 were malignant, and occurred in very young children (5 months, 19 months, and 3 years old) with underlying cancer predisposition syndromes (trisomy 18, Beckwith Wiedemann, Li Fraumeni respectively). These lesions showed only subtle washout (*n* = 2) or isoenhancement (*n* = 1) in the delayed phase. The 4th indeterminate lesion was a pathologically proven FNH-like lesion that occurred in an adolescent boy who was 12.5 years off therapy for medulloblastoma. There was concern that the patient was at risk for a second malignancy and the CEUS features were consistent with fibrolamellar HCC which has a predilection to occur in adolescent boys. Kong and colleagues reported hypoenhancement in the delayed phase in 10.7% (3/28) of FNH lesions in adults [26] and this pattern was seen in 2 of the 4 FNH lesions that were biopsied in our study. Others have speculated that this might be explained by retention of the contrast material in the damaged liver parenchyma that surrounds the FNH-like lesion [10]. Additional studies evaluating the CEUS enhancement patterns of FNH vs. FNH-like lesions are needed to better understand the expected findings in these conditions. All 4 false positive lesions in the more experienced radiologist’s (MBM) “unblinded” review showed contrast washout/hypoenhancement in the portal venous and delayed phases. Three of the 4 lesions were biopsied; 2 represented inflammatory or infectious processes and 1 showed hepatocytes in a liver that was iron overloaded. Another biopsied lesion, which was interpreted as benign on CEUS (i.e., no washout), also showed hepatocytes with iron overload. We are unable to explain the discrepancy in the CEUS enhancement patterns of lesions occurring in iron overloaded livers in our study subjects although it is possible that they represent developing regenerative nodules or FNH-like lesions. Importantly, among 5 biopsied infectious and inflammatory lesions in our study, 3 showed hypoenhancement in the portal venous and delayed phases. One, a microabscess/necrotizing granuloma (Fig. 3), showed substantial hypoenhancement in all three phases. Our findings are consistent with others who have shown that some benign lesions, such as scars, granulomas and inflammatory pseudotumors, may exhibit arterial phase iso or hypoenhancement and late phase hypoenhancement similar to malignant lesions [13, 27, 28]. Such lesions may also show a central area of non- or hypoenhancement depending on the degree of central liquefaction and replacement by purulent material [28, 29]. Taken together these findings suggest that the lack of arterial phase hyperenhancement or presence of arterial iso or hypoenhancement, coupled with washout/hypoenhancement in the portal venous or delayed phases, may be an important feature in distinguishing infectious or inflammatory from malignant etiologies of liver lesions.

While a direct comparison of CEUS to hepatocyte specific MRI was beyond the scope of this study, hepatocyte specific MRI has the benefit of allowing a more global assessment of the entire liver, may identify subtle lesions and can provide additional lesion characterization that aids in distinguishing benign from malignant etiologies [30–32]. Therefore, we recommend that hepatocyte specific MRI be performed when the results of CEUS are equivocal, are not in keeping with the clinical index of suspicion and when a malignant etiology is suspected (see Fig. 4). However, hepatocyte specific MRI must be interpreted with caution in the setting of iron overload. Because iron deposition in hepatocytes significantly decreases background liver signal, the typical hepatocyte specific contrast enhancement features of benign and malignant liver lesions may be significantly altered. In such cases, CEUS may be the preferred method to characterize liver lesions.

There are several limitations to this retrospective study. Although we had a limited sample size our study offers insight into a unique patient population that has not been previously reported. Additionally, histologic proof was available in only 19 of the 68 lesions, however, given that liver lesions are often found incidentally and are typically of low malignant potential, it would be unethical to perform biopsy on all patients. While general patterns of ultrasound contrast-enhancement are established for benign and malignant lesions, the patterns of treated metastatic disease are not well understood. It is possible that some of our cases determined to be benign based on clinical follow-up reflect treated metastatic disease that was not previously detected. However, a benign lesion vs. treated metastatic disease may carry the same functional outcome. The ultrasound contrast agent used was not uniform throughout the study although most lesions (90%) were imaged with one agent. There were technical challenges during examination of very young patients, some of whom had difficulty holding still, and breathing motion sometimes made it difficult to maintain the lesion within the field of view. However, a radiologist was present for every examination and repeat injections were given, as needed, to improve diagnostic confidence. Another limitation is that the experienced study radiologists may have had some recall bias after being unblinded to the subject’s clinical history. We attempted to mitigate this by allowing sufficient time to pass between the clinical exam and the review for study purposes.

## Conclusion

We have shown that CEUS is a reliable and accurate method of distinguishing benign from malignant liver lesions in most patients treated for childhood malignancies. Perhaps most importantly, it can reduce the anxiety surrounding the discovery of such lesions in this patient population and facilitates prompt clinical management. We acknowledge that there are challenges to the generalizability of this modality and to mitigate these we provide a clinical management algorithm that considers CEUS findings together with relevant clinical features. Several studies investigating the value of quantitative CEUS in the assessment of FLLs show promise in improving the ability to provide specific benign diagnoses and this area warrants further investigation [33, 34]. We propose a call to action to increase the use of CEUS in pediatric oncology and adult survivors of childhood cancer to improve our understanding of and confidence in this low risk, high-yield modality for assessment of liver lesions.

## Data Availability

All data generated or analyzed during the study are included in this published article.

## Abbreviations

CT: Computed tomography
MRI: Magnetic resonance imaging
US: Ultrasound
CEUS: Contrast enhanced ultrasound
FLL: Focal liver lesion
FNH: Focal nodular hyperplasia
