# Correction: Contrast enhanced ultrasound of liver lesions in patients treated for childhood malignancies

**DOI:** 10.1186/s40644-024-00785-6

**Published:** 2024-10-14

**Authors:** Ayatullah G. Mostafa, Zachary Abramson, Mina Ghbrial, Som Biswas, Sherwin Chan, Himani Darji, Jessica Gartrell, Seth E. Karol, Yimei Li, Daniel A. Mulrooney, Tushar Patni, Tarek M Zaghloul, M. Beth McCarville

**Affiliations:** 1 Department of Diagnostic Imaging, St. Jude Children’s Research Hospital, MS 220, 262 Danny Thomas Place, Memphis, TN 38105 USA; 2 Department of Diagnostic Imaging, Cairo University, Cairo, Egypt; 3 Pediatric Radiology Department, Le Bonheur Children’s Hospital, Memphis, TN USA; 4 Department of Radiology, Children’s Mercy Hospital, 2401 Gillham Rd, Kansas City, MO 64108 USA; 5 Department of Biostatistics, St. Jude Children’s Research Hospital, 262 Danny Thomas Place, Memphis, TN 38105 USA; 6 Department of Oncology, St. Jude Children’s Research Hospital, MS 260, 262 Danny Thomas Pl, Memphis, TN 38105 USA; 7 Department of Surgery, St. Jude Children’s Research Hospital, MS 133, 262 Danny Thomas Place, Memphis, TN 38105 USA; 8 Department of Surgery, National Cancer Institute, Cairo University, Cairo, Egypt

**Correction: Cancer Imaging (2024) 24:115**

10.1186/s40644-024-00750-3

Following publication of the original article [1], we were notified that two first authors need to be marked as co-first authors and that the initial G. needs to be add to the first author’s name.

Additionally, originally published affiliation no 2 (Rutgers New Jersey Medical School, Newark, USA), linked to Prof. Mostafa should actually read as Department of Diagnostic Imaging, Cairo University, Cairo, Egypt and originally published affiliation 8 (Quantitative Sciences Unit, Stanford University, 3180 Porter Drive, Palo Alto, CA 94304, USA), linked to Prof. Tarek M Zaghloul should be entirely removed.

The original article has been corrected.
